# A new lymph node ratio-based staging system for rectosigmoid cancer: a retrospective study with external validation

**DOI:** 10.1097/JS9.0000000000000546

**Published:** 2023-07-19

**Authors:** Chao Zhang, Shutao Zhao, Xudong Wang, Dacheng Wen

**Affiliations:** Department of Gastrointestinal Nutrition and Hernia Surgery, The Second Hospital of Jilin University, Changchun, People’s Republic of China

**Keywords:** lymph node ratio, lymph node, OS, rectosigmoid cancer, stage

## Abstract

**Background:**

This study evaluated the clinical value of a new American Joint Committee on Cancer (AJCC) tumor node metastasis (TNM) staging prediction model based on lymph node ratio (LNR) in rectosigmoid cancer (RSC).

**Methods:**

The analysis included 1444 patients with nonmetastatic RSC diagnosed pathologically between 2010 and 2016 who were collected from the National Cancer Institute Surveillance, Epidemiology, and Results database. The AJCC N-stage was redefined according to the LNR cutoff point, and the ability of the new staging system to predict prognosis was compared with that of the AJCC TNM staging system. Data from 739 patients from our hospital were used for external validation.

**Results:**

According to the number of examined lymph nodes and LNR, the N stage was divided into five groups (LNR0–5). The 5-year OS of patients divided according to the new T lymph node ratio M (TLNRM) staging into stage I (T1LNR1, T1LNR2), IIA (T1LNR3, T2LNR1, T2LNR2, T2LNR3, T1LNR4, T3LNR1), IIB (T2LNR4), IIC (T3LNR2, T4a LNR1, T1LNR5), IIIA (T3LNR3, T2LNR5, T4b LNR1, T4a LNR2, T3LNR4), IIIB (T3LNR5, T4a LNR3, T4a LNR4, T4b LNR2), and IIIC (T4b LNR3, T4a LNR5, T4b LNR4, T4b LNR5) was significantly different (*P*<0.05). Decision curve analysis showed that the net income of the new TLNRM staging system for different decision thresholds was higher than the prediction line of the traditional eighth TNM staging system. The smaller Akaike information criterion and Bayesian information suggested that the new staging system had a higher sensitivity for predicting prognosis than the traditional staging system. TLNRM II and III patients benefited from adjuvant chemotherapy, while adjuvant chemotherapy did not improve the prognosis of TNM II patients. These findings were confirmed by the external validation data.

**Conclusion:**

The new TLNRM staging system was superior to the eighth edition AJCC staging system for staging and predicting the prognosis of patients with RSC and may become an effective tool in clinical practice.

## Background

Highlights

- This study established the clinical value of a new American Joint Committee on Cancer tumor node metastasis staging prediction model.
- The new T lymph node ratio M staging system was superior to the traditional eighth tumor node metastasis staging system.
- T lymph node ratio M II and III rectosigmoid cancer patients benefited from adjuvant chemotherapy.

According to the 2020 global cancer statistics, rectal cancer ranks 8th in incidence and 9th in mortality^1^. There is no unified definition of rectosigmoid cancer (RSC), and the current theory defines it as surgical rectum^2^. Therefore, the staging and treatment of RSC are based on the established protocols for rectal cancer, underscoring the need to develop specific staging and treatment methods for RSC to provide more accurate treatments for patients with this disease. At present, the most commonly used clinical staging prediction model for rectal cancer is the eighth version of the tumor node metastasis (TNM) classification system proposed by the American Joint Committee on Cancer (AJCC)^3–5^. However, the N stage, according to AJCC staging is based solely on the number of positive lymph nodes (PLN). The number of examined lymph nodes (ELN) by surgery is positively correlated with the number of PLN, and the number of ELN during surgery is a key factor for evaluating the efficacy of radical resection of rectal cancer.

Considering the specificity of the location of RSC and the shortcomings of N staging, we propose a new staging method based on the positive lymph node ratio (LNR) to improve the traditional staging^6–8^. LNR is the ratio of the number of PLN to the total number of ELN. Its ability to predict the prognosis of patients is not affected by the number of ELN. The independent predictive ability of LNR in colorectal cancer has been demonstrated. The establishment of new LNR grouping variables will improve the prognostic stratification of RSC patients^9–11^.

In this study, RSC data from the Surveillance, Epidemiology, and End Results (SEER) Database were used to divide N stage patients into five groups according to LNR. The sensitivity of the new T lymph node ratio M (TLNRM) staging system was compared with that of traditional staging, and the accuracy of the patient’s prognostic evaluation was assessed, which selected the population that could most benefit from adjuvant chemotherapy (ACT). The advantages of the new TLNRM staging were verified using external data.

## Methods

### Patient cohort

SEER * Stat (version 8.4.0) software was used to search 1444 cases of nonmetastatic RSC diagnosed between 2010 and 2016. The inclusion criteria were as follows: patients with RSC diagnosed by pathology (ICD-O-3: C19.9), complete follow-up and survival data, no neoadjuvant radiotherapy and chemotherapy, underwent radical laparoscopic surgery, and primary RSC. The variables included in this study included age, sex, race, grade, size, T stage, ELN, PLN, and survival information. Patients with missing information were excluded. The external validation data were obtained from 739 patients with nonmetastatic RSC who underwent surgery in our department between 2010 and 2017. Advanced patients received ACT with the XELOX regimen. The staging of all patients was adjusted to the TNM staging, eighth edition. The work has been reported in line with the strengthening the reporting of cohort, cross-sectional and case–control studies in surgery (STROCSS) criteria.

### Statistical analysis

The distribution of clinical and pathological factors was assessed. When LNR=0, X-tile software was used to divide the patients into two groups: LNR1 (*n*=133) (ELN 0–4) and LNR2 (*n*=327) (ELN 5–12). When LNR greater than 0, X-tile software was used to select two cutoff values to divide the patients into three groups: LNR3 (*n*=427; ratio 0.08–0.22), LNR4 (*n*=198; ratio 0.25–0.45), and LNR5 (*n*=359; ratio 0.50–1.00). An improved TLNRM staging system was established by replacing N staging from the eighth version of AJCC staging with the corresponding LNR staging.

The patients were divided into 25 groups (T1-4bLNR1-5M0). The hazard ratio (HR) of T1LNR1M0 was defined as 1, which was sorted from low to high. The two adjacent groups were tested by the log-rank of OS. Six groups with large differences were selected from 24 *χ* ^2^ values, and the 25 groups were divided into 7 stages.

The effectiveness of the TLNRM staging system was compared with that of the eighth edition AJCC staging system in terms of prognosis discrimination. The discrimination ability of the two staging systems was quantified using the Akaike information criterion (AIC) and Bayesian information (BIC)^12^. The clinical net benefits were evaluated by decision curve analysis (DCA) and compared with those of the eighth edition of the AJCC staging system^13^. The *χ* ^2^-test was used to evaluate the prognostic homogeneity of the two staging systems (the greater the likelihood ratio, the better the prognostic homogeneity of the staging system), and survival curves and COX analysis were used to analyze the prognosis of patients in different stages. SPSS 24.0 and R language (version 4.0.0) were used for all statistical analyses, and *P*<0.05 was considered as statistically significant.

## Results

### Patient demographics

**Figure 1:**
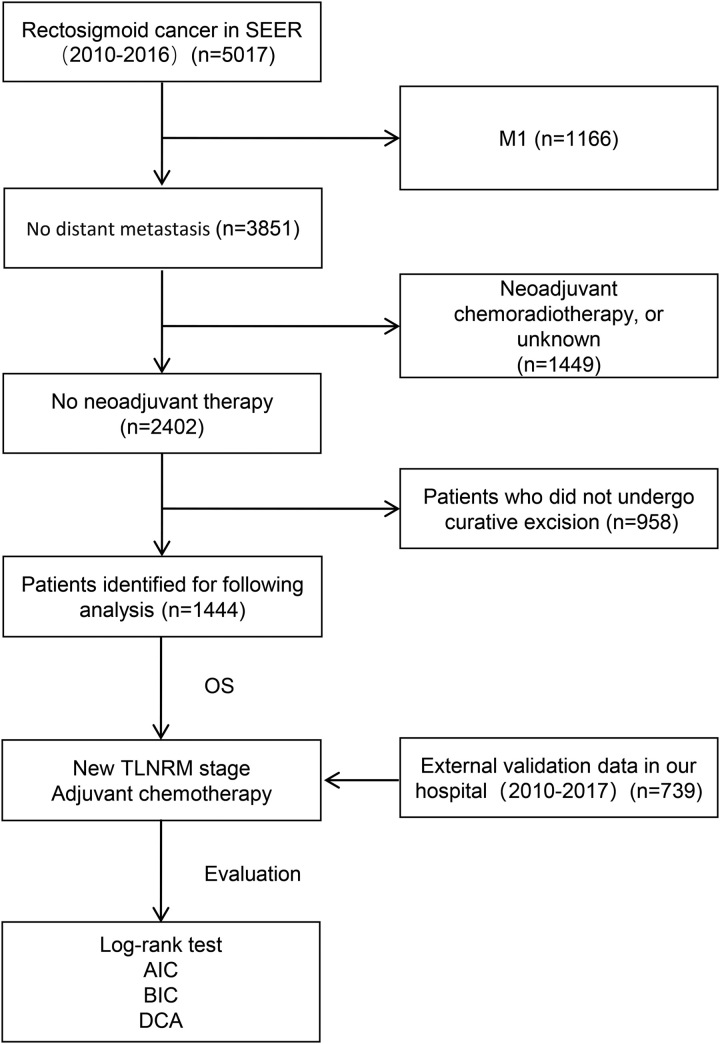
Flowchart of the selection process of included patients. AIC, Akaike information criterion; BIC, Bayesian information; DCA, decision curve analysis; SEER, Surveillance, Epidemiology, and End Results.

A total of 1444 patients with RSC were included according to the inclusion criteria (Fig. 1). There were 846 patients (58.6%) greater than or equal to 65-year-old and 719 patients (49.8%) with T3-4b. The median number of ELN was 9, and the median number of PLN was 2 (Table 1). The median survival of patients was 85 months (0–191) and the number of deaths was 764 (52.9%).

**Table 1:** Characteristics of patients.

Variable	Total [*n* (%)]	Training [*n* (%)]	External [*n* (%)]	*P*
Age		1444	739	<0.001
<65		598 (41.4)	506 (68.5)	
≥65		846 (58.6)	233 (31.5)	
Sex				<0.001
Male		784 (54.3)	527 (71.3)	
Female		660 (45.7)	212 (28.7)	
Race				<0.001
White		1108 (76.7)	0 (0.0)	
Black		99 (6.9)	0 (0.0)	
API		217 (15.0)	0 (0.0)	
Other		20 (1.4)	739 (100.0)	
Grade				<0.001
Well/moderately		1200 (83.1)	314 (42.5)	
Poorly/undifferentiated		166 (11.5)	363 (49.1)	
Unknown		78 (5.4)	62 (8.4)	
Size (cm)				<0.001
<3		458 (31.7)	133 (18.0)	
≥3		781 (54.1)	606 (82.0)	
Unknown		205 (14.2)	0 (0.0)	
T stage				<0.001
T1		400 (27.7)	128 (17.3)	
T2		325 (22.5)	98 (13.3)	
T3		616 (42.7)	233 (31.5)	
T4a		68 (4.7)	189 (25.6)	
T4b		35 (2.4)	91 (12.3)	
ELN count, median		9	10	<0.001
Positive ELN count, median		2	1	<0.001

API, Asian/Pacific Islander; ELN, examined lymph nodes.

### A new LNR staging system

**Figure 2:**
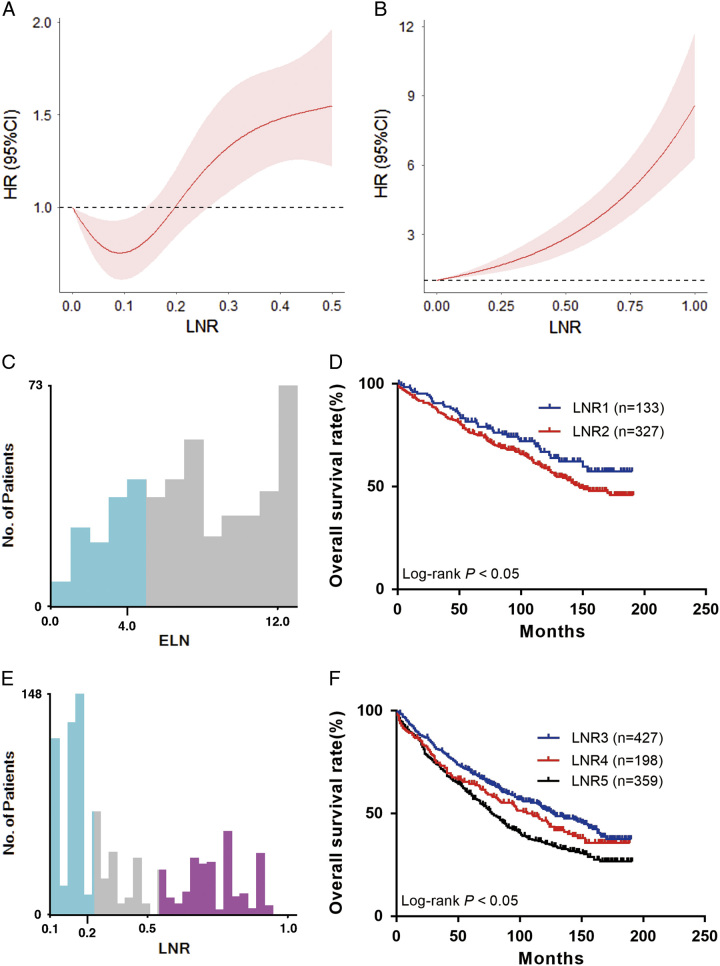
(A) hazard estimates of death from training set; (B) hazard estimates of death from external set; (C) the optimal cutoff value for lymph node ratio =0; (D) overall survival for patients in two subgroups; (E) the optimal cutoff value for lymph node ratio >0; (F) overall survival for patients in three subgroups. LNR, lymph node ratio.

**Figure 3:**
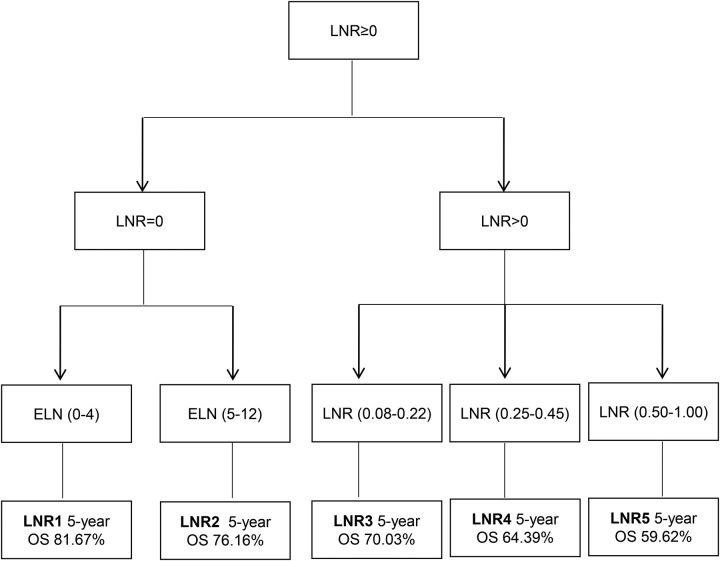
The new lymph node ratio staging system. ELN, examined lymph nodes; LNR, lymph node ratio.

**Figure 4:**
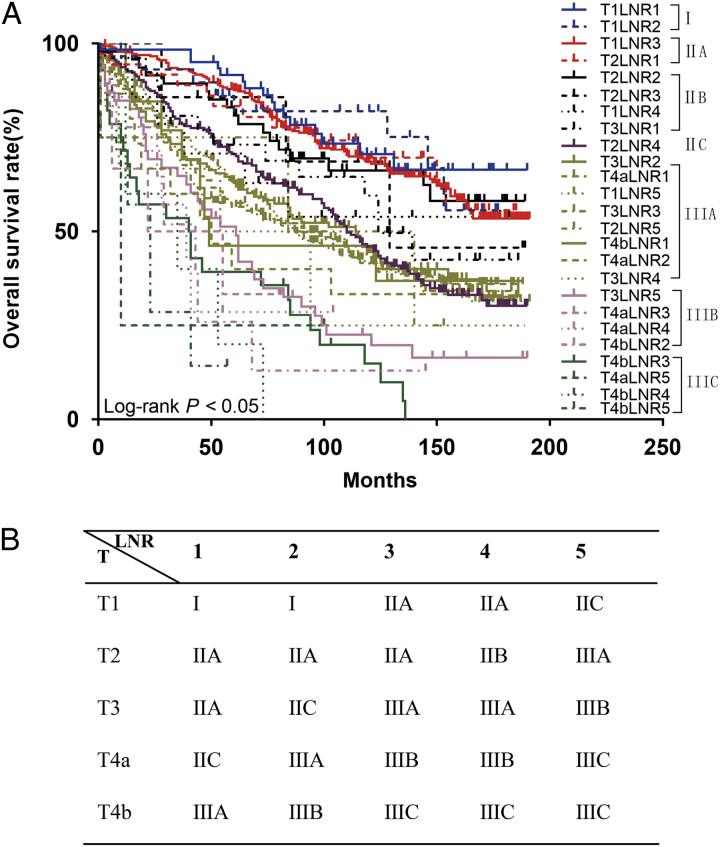
(A) The Kaplan–Meier curves of overall survival for patients in our new lymph node ratio staging system; (B) the new TLNRM staging system. LNR, lymph node ratio.

**Figure 5:**
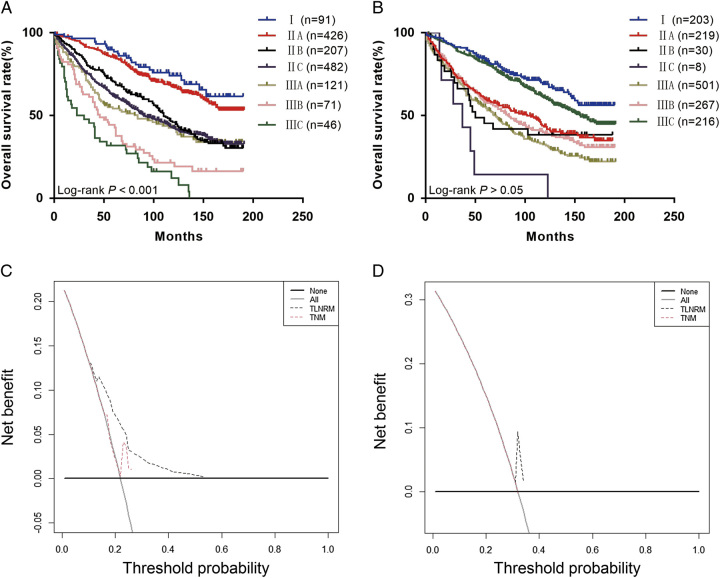
(A) The Kaplan–Meier curves of overall survival (OS) for TLNRM staging system; (B) the Kaplan–Meier curves of OS for traditional tumor node metastasis (TNM) staging system; (C) TLNRM stage were compared to the TNM stage in terms of 3-year OS in our decision curve analysis; (D) TLNRM stage were compared to the TNM stage in terms of 5-year OS in our decision curve analysis.

A higher LNR was associated with a worse prognosis (Fig. 2A). Patients were divided into five groups according to the LNR value. When LNR=0, X-tile software divided the patients into two groups: LNR1 (*n*=133) (ELN 0–4) and LNR2 (*n*=327) (ELN 5–12) (ELN 0–4) (Fig. 2C, D), and when LNR greater than 0, X-tile software selected two optimal cutoff values to divide the patients into three groups LNR3 (*n*=427; ratio 0.08–0.22), LNR4 (*n*=198; ratio 0.25–0.45), and LNR5 (*n*=359; ratio 0.50–1.00) (Fig. 2E, F), the 5-year survival rates of the LNR1–5 were 81.67, 76.16, 70.03, 64.39, and 59.62%, respectively (Fig. 3). According to the eighth edition of staging, patients in the traditional N staging corresponding to LNR1–5 were divided into 25 groups. The HR of T1LNR1M0 was defined as 1, and the HR value was calculated from low to high, with the highest being T4b LNR5M0 (HR=11.976; 95% CI: 4.669–30.721; *P*<0.001; Table 2). Survival analysis was performed for two adjacent stages, and *χ* ^2^ values were calculated. Six high values were selected as cutoff points (0.180, 0.870, 0.374, 0.142, 0.260, and 0.207). Patients were divided into seven stages, stage I (T1LNR1, T1LNR2), IIA (T1LNR3, T2LNR1, T2LNR2, T2LNR3, T1LNR4, T3LNR1), IIB (T2LNR4), IIC (T3LNR2, T4a LNR1, T1LNR5), IIIA (T3LNR3, T2LNR5, T4b LNR1, T4a LNR2, T3LNR4), IIIB (T3LNR5, T4a LNR3, T4a LNR4, T4b LNR2), and IIIC (T4b LNR3, T4a LNR5, T4b LNR4, T4b LNR5) (Fig. 4), and the 5-year survival rates of the seven stages were 88.58, 84.96, 70.57, 61.89, 54.17, 44.29, and 31.82%, respectively, with a statistically significant difference (*P*<0.05; Fig. 5A). The 5-year survival rates from stage I to IIIC of traditional TNM staging were 83.31, 59.93, 45.30, 14.29, 81.58, 55.26, and 58.13%, respectively, and the difference was not statistically significant (*P*>0.05; Fig. 5B).

**Table 2:** The COX analyses of factors associated with overall survival in the training set.

	Cox regression		Log-rank (Mantel–Cox)	
Variable	HR (95% CI)	*P*	*χ* ^2^	*P*
Stage I				
T1LNR1	1			
T1LNR2	1.052 (0.450–2.459)	0.906	0.010	0.921
Stage IIA				
T1LNR3	1.273 (0.752–2.155)	0.369	0.180	0.671
T2LNR1	1.326 (0.639–2.758)	0.449	0.011	0.917
T2LNR2	1.443 (0.736–2.829)	0.286	0.050	0.823
T2LNR3	1.587 (0.462–5.448)	0.463	0.050	0.823
T1LNR4	1.935 (0.757–4.945)	0.168	0.069	0.792
T3LNR1	1.979 (0.952–4.115)	0.068	0.001	0.978
Stage IIB				
T2LNR4	2.573 (1.529–4.330)	<0.001	0.870	0.351
Stage IIC				
T3LNR2	2.798 (1.623–4.823)	<0.001	0.374	0.541
T4a LNR1	2.847 (1.639–4.948)	<0.001	0.023	0.880
T1LNR5	2.949 (1.764–4.930)	<0.001	0.056	0.813
Stage IIIA				
T3LNR3	3.057 (1.733–5.392)	<0.001	0.142	0.706
T2LNR5	3.111 (0.906–10.680)	0.071	0.005	0.943
T4b LNR1	3.252 (1.437–7.361)	0.005	0.008	0.930
T4a LNR2	3.464 (1.571–7.635)	0.002	0.021	0.884
T3LNR4	3.807 (1.273–11.391)	0.017	0.015	0.902
Stage IIIB				
T3LNR5	5.166 (2.886–9.248)	<0.001	0.260	0.610
T4a LNR3	5.874 (1.962–17.589)	0.002	0.014	0.907
T4a LNR4	6.381 (2.334–17.442)	<0.001	0.012	0.912
T4b LNR2	6.617 (2.719–16.102)	<0.001	0.005	0.943
Stage IIIC				
T4b LNR3	7.307 (3.951–13.514)	<0.001	0.207	0.649
T4a LNR5	8.792 (2.559–30.202)	0.001	0.092	0.762
T4b LNR4	9.773 (3.573–26.727)	<0.001	0.099	0.753
T4b LNR5	11.967 (4.669–30.721)	<0.001	0.331	0.565

LNR, lymph node ratio.

### Comparison of the prognostic effectiveness of the two staging systems

The AIC (10354.11) and BIC (10358.75) of traditional TNM were higher than the AIC (10220.55) and BIC (10225.19) of TLNRM staging. The χ^2^-test of the likelihood ratio of traditional TNM (133.583) was lower than that of TLNRM staging (196.090) (Table 3). The DCA curve showed that the TLNRM staging system had higher net benefits than the traditional TNM staging system (Fig. 5C, D). These findings indicated that the TLNRM staging system was superior to the eighth version of the AJCC staging system.

**Table 3:** Effectiveness of the two staging systems for predicting prognosis.

Stage	Likelihood ratio (*χ* ^2^)	AIC	BIC	*P*
TNM (Training)	133.583	10 354.11	10 358.75	<0.001
TLNRM (Training)	196.090	10 220.55	10 225.19	<0.001
TNM (External)	318.688	4314.199	4318.099	<0.001
TLNRM (External)	390.910	4219.002	4222.901	<0.001

AIC, Akaike information; BIC, Bayesian information criterion.

### Testing the efficiency of ACT for patients in different stages

**Figure 6:**
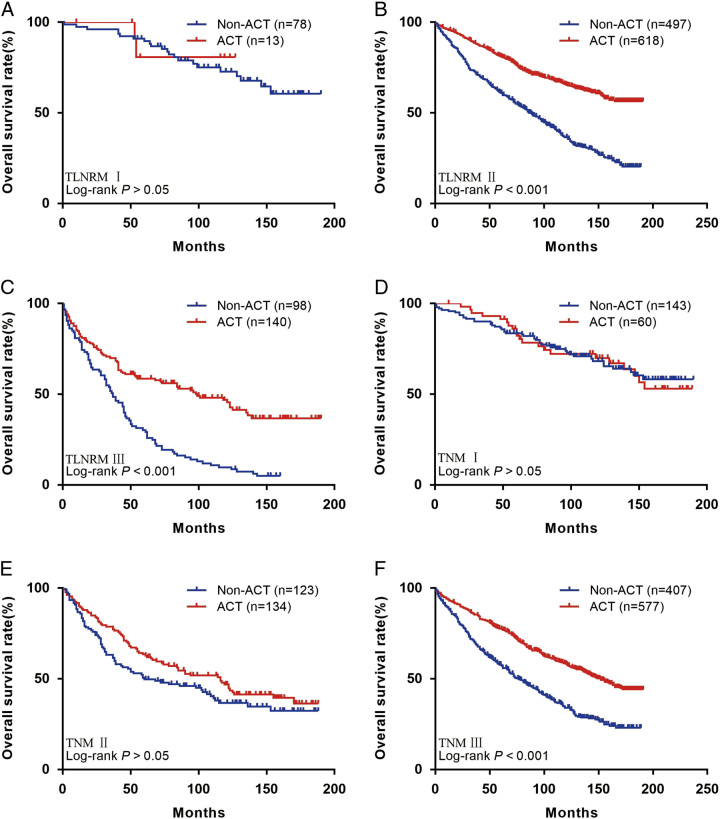
(A) Overall survival (OS) for patients with or without adjuvant chemotherapy (ACT) in TLNRM stage I group; (B) OS for patients with or without ACT in TLNRM stage II group; (C) OS for patients with or without ACT in TLNRM stage III group; (D) OS for patients with or without ACT in TNM stage I group; (E) OS for patients with or without ACT in TNM stage II group; (F) OS for patients with or without ACT in tumor node metastasis stage III group. ACT, adjuvant chemotherapy; TNM, tumor node metastasis.

We further investigated the benefit of ACT in patients with different stages. The results showed that patients in the TLNRM I group did not benefit from ACT (HR: 0.75, 95% CI: 0.21–2.74, *P*>0.05; Fig. 6A). Patients in the TLNRM II (HR: 0.41, 95% CI: 0.34–0.49, *P*<0.001; Fig. 6B) and III (HR: 0.42, 95% CI: 0.30–0.58, *P*<0.001; Fig. 6C) group benefit from ACT. Patients in the TNM I (HR: 1.02, 95% CI: 0.61–1.72, *P*>0.05; Fig. 6D) and II (HR: 0.76, 95% CI: 0.55–1.06, *P*>0.05; Fig. 6E) group did not benefit from ACT. Patients in the TNM III (HR: 0.51, 95% CI: 0.43–0.62, *P*<0.001; Fig. 6F) group benefit from ACT.

### TLNRM staging system in the external validation group

**Figure 7:**
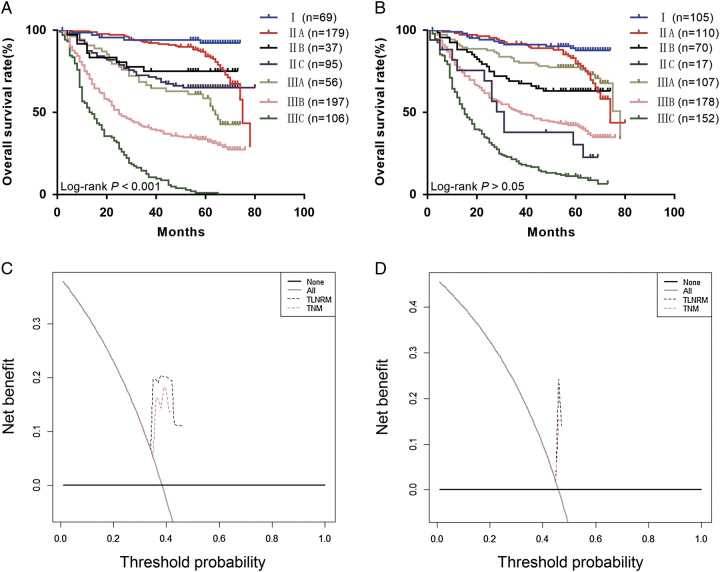
(A) The Kaplan–Meier curves of overall survival (OS) for TLNRM staging system in our external set; (B) the Kaplan–Meier curves of OS for traditional tumor node metastasis (TNM) staging system in our external set; (C) TLNRM stage were compared to the TNM stage in terms of 3-year OS in our decision curve analysis in our external set; (D) TLNRM stage were compared to the TNM stage in terms of 5-year OS in our decision curve analysis in our external set. TNM, tumor node metastasis.

According to the same inclusion criteria, 739 postoperative patients with RSC were collected from our hospital; of these, 513 (69.4%) were T3-4b patients. The median number of ELN was 10, and the median number of PLN was 1. The median survival period of the patients was 56 months (0–80), and the number of deaths was 365 (49.4%). According to the grouping of the SEER database, the patients were divided into seven stages as follows: I, IIA, IIB, IIC, IIIA, IIIB, and IIIC, and the 5-year survival rates were 92.32, 84.07, 75.00, 65.14, 58.66, 33.66, and 0.0%, respectively. The difference was statistically significant, *P*<0.05 (Fig. 7A). The 5-year survival rates from stage I to III of traditional TNM staging were 87.79, 79.60, 63.06, 30.25, 77.36, 42.11, and 11.14%, and the difference was not statistically significant (*P*>0.05; Fig. 7B).

The AIC (4314.199) and BIC (4318.099) of traditional TNM were higher than the AIC (4219.002) and BIC (4222.901) of TLNRM staging. The χ^2^-test of the likelihood ratio of traditional TNM (318.688) was lower than that of TLNRM staging (390.910) (Table 3). The DCA curve showed that the TLNRM staging system had higher net benefits than the traditional TNM staging system (Fig. 7C, D). The external validation data confirmed that the TLNRM staging system was superior to the eighth version of the AJCC staging system.

**Figure 8:**
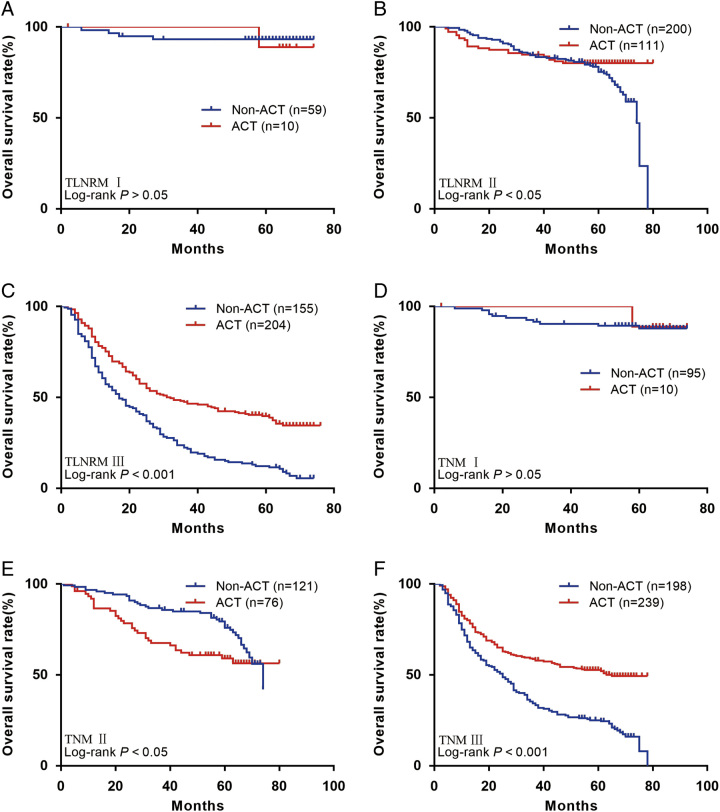
(A) Overall survival (OS) for patients with or without adjuvant chemotherapy (ACT) in TLNRM stage I group in our external set; (B) OS for patients with or without ACT in TLNRM stage II group in our external set; (C) OS for patients with or without ACT in TLNRM stage III group in our external set; (D) OS for patients with or without ACT in tumor node metastasis (TNM) stage I group in our external set; (E) OS for patients with or without ACT in TNM stage II group in our external set; (F) OS for patients with or without ACT in TNM stage III group in our external set. ACT, adjuvant chemotherapy; TNM, tumor node metastasis.

We further investigated the benefit of ACT in patients with different stages (in the external validation group. The results showed that patients in the TLNRM I group did not benefit from ACT (HR: 1.50, 95% CI: 0.12–18.41, *P*>0.05; Fig. 8A). Patients in the TLNRM II (HR: 0.60, 95% CI: 0.39–0.93, *P*<0.05; Fig. 8B) and III (HR: 0.49, 95% CI: 0.38–0.63, *P*<0.001; Fig. 8C) group benefit from ACT. Patients in the TNM I (HR: 0.86, 95% CI: 0.13–5.86, *P*>0.05; Fig. 8D) and II (HR: 1.65, 95% CI: 1.00–2.72, *P*<0.05; Fig. 8E) group did not benefit from ACT. Patients in the TNM III (HR: 0.48, 95% CI: 0.38–0.61, *P*<0.001; Fig. 8F) group benefit from ACT.

## Discussion

The AJCC system stages patients according to the invasion depth of the primary tumor (T), the regional ELN (N), and whether there is distant metastasis^14–16^. AJCC N staging is based solely on the number of PLN. In addition, the technological skills of the surgeon, the monitoring experience of the pathologist, and other unavoidable circumstances affect the number of ELN, which may lead to the stage migration of patients^17,18^. RSC occurs in the spiral mucosa of the sigmoid colon, which belongs to the colon anatomically. However, the rectum and RSC in the peritoneal retraction share a common vascular system^19,20^, which is similar to rectal cancer in prognosis. Some scholars believe that this tumor should be classified as rectal cancer, and its staging also mainly refers to rectal cancer^21^. Considering these issues, we attempted to develop a better staging index to replace the N staging from the AJCC TNM staging of RSC.

LNR is defined as the number of PLN divided by the total number of ELN. LNR is an important prognostic factor in lung cancer, oral cancer, esophageal cancer, and other malignant tumors. LNR-based staging may be more accurate than the traditional N staging system for evaluating prognosis^22–27^. The number of ELN in the right colon cancer was higher than that in the left. Shinto *et al.* ^6^ showed that the LNR of the right and left sides in stage III colon cancer was 0.16 and 0.22, respectively, and the survival of patients with low LNR was better than that of those with high LNR in both left and right colon cancers. Ceelen *et al.*^28^ analyzed 16 studies and found that LNR was an independent prognostic factor of OS (HR=2.36; 95% CI: 2.14–2.61; *P*<0.05) in patients with stage III colorectal cancer, and the stratification effect on prognosis is better than that of traditional N staging. In patients with stage III rectal cancer, the optimal cutoff value of LNR is 0.257, whereas the AUC of the traditional N stage is 0.669, with a sensitivity of 43% and a specificity of 86%^29^. Macedo *et al.* ^30^ found that LNR was an independent prognostic factor for colorectal cancer patients (HR=1.250; 95% CI: 1.077–1.450; *P*<0.01), and its prognostic value is higher than that of the traditional N stage. LNR can simultaneously evaluate the number of PLN and the number of ELN, thereby reducing the staging bias caused by insufficient ELN. However, the value of LNR in RSC has not been reported to date. Here, we evaluated the prognostic significance of LNR in RSC.

Because the cutoff values of LNR differ among colorectal cancer studies^31–34^, we divided patients into five groups using an X-tile according to an LNR ratio of 0 or greater than 0, which had good differentiation potential. Combined with the T stage, we divided patients into seven stages. A higher stage was associated with a worse prognosis. Stage II colon cancer patients with less than 12 ELN are high-risk patients, and chemotherapy is highly recommended for these patients. Stage bias may have occurred in these patients, and their prognosis is similar to that of stage IIIB patients^35^. In the present study, T4a LNR1 migrated to stage IIC, and T4b LNR1 migrated to stage IIIA, suggesting that the proposed N staging was an effective method for avoiding stage bias. Compared with the eighth edition of the AJCC staging system, the new TNM staging system was superior for determining the 5-year prognosis of patients (*P*<0.05). The higher accuracy of the new TNM staging system for predicting prognosis could be attributed to the additional prognostic value of LNR. We also observed that the heterogeneity between the new TNM staging systems was relatively low as indicated by AIC, BIC, and *χ* ^2^, which significantly improved patient stratification and the prognosis prediction efficiency. The new staging system thus provided a reliable, practical, standardized, and personalized method for oncologists, which will improve the design of treatments and the efficacy of follow-up evaluations. Finally, for patients with insufficient ELN detection, the new staging system reduced the risk of insufficient treatment or follow-up.

We further verified the patients who could really benefit from ACT in the new TLNRM stage. The results showed that patients in the TLNRM I group did not benefit from ACT, patients in the TLNRM II and III group benefit from ACT. Patients with advanced colorectal cancer could benefit from ACT, which is consistent with our results^36–38^. However, traditional TNM staging showed that patients with stage II RSC could not benefit from ACT. It showed that there might be leading to stage migration in the traditional TNM stage, and our TLNRM stage could correctly guide the ACT of postoperative RSC patients.

The present study had several limitations. First, a decreased number of ELN due to various reasons, such as a gap in technology, the inexperience of surgeons, or the small size of lymph nodes in the specimen, will have an impact on the total number of ELN, which is an unavoidable problem for the SEER database and our own database. Second, this was a retrospective study, and some patients were not included because of data loss, which may lead to bias. Third, the new staging system is not applicable to patients with stage IV RSC. Fourth, the definition of RSC in our center and the SEER database may have inconsistent standards. Fifth, there are certain differences between ELN, PLN, and median survival in our center and the SEER database, which may lead to certain bias. Sixth, our external validation is single center. The staging and treatment of RSC are controversial. Stratifying patients using LNR staging will help with the design of individualized treatments, which highlights the importance of this article^39^.

## Conclusion

The new TNM staging system proposed can lead to improved staging and prognosis prediction in patients with RSC. It showed a superior predictive ability, which is important for the clinical management of patients with RSC.

## Ethical approval

This study is a retrospective cohort study in SEER database. All procedures performed in studies involving human participants were in accordance with the ethical standards of the Institutional and National Research Committee and with the 1964 Helsinki Declaration and its later amendments or comparable ethical standards. The SEER Program collects data from population-based cancer registries with anonymous information. The SEER is a publicly available database, thus no ethical approval is required.

## Sources of funding

National Natural Science Foundation of China (No 82203801); Department of Science and Technology of Jilin Province (No YDZJ202202CXJD047; YDZJ202301ZYTS037). The Bethune Project (2023B12).

## Author contribution

X.W.: conceptualization, methodology, software; C.Z.: data curation, writing – original draft preparation; D.W.: data curation, investigation; S.Z.: visualization, investigation, super-vision, and validation.

## Conflicts of interest disclosure

The authors declare no conflicts of interest.

## Research registration unique identifying number (UIN)

1. Name of the registry: Chinese Clinical Trial Registry.
2. Unique identifying number or registration ID: ChiCTR2100050554.
3. Hyperlink to your specific registration (must be publicly accessible and will be checked):http://www.chictr.org.cn/showprojen.aspx?proj=132614

## Guarantor

Xudong Wang and Dacheng Wen accept full responsibility for the work. Chao Zhang had access to the data.

## Data availability statement

The raw data of this study are derived from the SEER database, which is a publicly available database. All detailed data included in the study are available upon request by contact with the corresponding author.

## Provenance and peer review

Not commissioned, externally peer-reviewed.
